# Comparative Predictive Performance of Upper Gastrointestinal Bleeding Risk Scores and the CALLY Index in Geriatric Patients

**DOI:** 10.3390/diagnostics16162664

**Published:** 2026-08-20

**Authors:** Muge Gul Gulecoglu Onem, Soner Onem

**Affiliations:** 1 Department of Clinical Biochemistry, Samsun City Hospital, Samsun 55080, Turkey; mugegl@hotmail.com; 2 Department of Gastroenterology, Faculty of Medicine, Dokuz Eylul University, Izmir 35330, Turkey

**Keywords:** upper gastrointestinal hemorrhage, elderly, geriatric, CALLY index, Glasgow-Blatchford score, Rockall score, risk stratification

## Abstract

**Background/Objectives**: Elderly patients with acute upper gastrointestinal bleeding (UGIB) are a high-risk group with significant morbidity and mortality. Various risk scoring systems have been devised to predict clinical outcome in UGIB, but their effectiveness in geriatric patients is unclear. The purpose of the present study was to evaluate the predictive performance of established UGIB risk scores in older adults and to compare the predictive performance of the C-reactive protein–albumin–lymphocyte (CALLY) index with conventional UGIB risk scores. **Methods**: This study was a retrospective single-center analysis of a cohort of patients aged ≥65 years presenting with acute non-variceal upper gastrointestinal bleeding (UGIB) who had esophagogastroduodenoscopy from January to December 2025. Scores for Glasgow-Blatchford Score (GBS), Rockall, AIMS65, ABC, MAP(ASH), CANUKA, T-score, and CALLY index were calculated. The primary management endpoint was the requirement for endoscopic hemostatic treatment. Secondary outcomes were blood transfusion necessity, rebleeding, intensive care unit (ICU) admission, length of hospital stay, and 30-day death. The predictive performance of each scoring system was assessed by receiver operating characteristic (ROC) curve analysis. **Results**: We enrolled 113 patients (mean age 77.7 ± 8.3 years; 59.3% male), of whom 42 (37.2%) required endoscopic hemostatic treatment. No single risk score consistently outperformed the others across all clinical outcomes. GBS showed the highest observed AUC for predicting the need for transfusion (AUC = 0.892), whereas Rockall showed the highest observed AUC for rebleeding (AUC = 0.739) and ICU admission (AUC = 0.668). In the exploratory mortality analysis based on seven deaths, the directionally corrected AUC was 0.846 for CALLY, 0.756 for MAP(ASH), and 0.742 for CANUKA; no pairwise AUC comparisons were performed for this outcome. The CALLY index showed modest discrimination for endoscopic hemostatic therapy (AUC = 0.664). In the exploratory rebleeding analysis, CALLY yielded an AUC of 0.724; however, this estimate was based on only eight events. Lower CALLY levels were associated with increased 30-day mortality, whereas the CALLY index was not significantly associated with transfusion requirements or length of hospital stay. After correction for multiple comparisons, CALLY showed significantly lower discriminatory performance for transfusion requirement than GBS, T-score, CANUKA, and MAP(ASH), whereas no significant differences were observed for endoscopic intervention or ICU admission. **Conclusions**: No single risk score was able to predict all clinically important outcomes in older adults with acute UGIB. Conventional scoring systems remained useful for several established clinical outcomes, whereas the CALLY index showed modest discrimination for endoscopic hemostatic therapy. Its findings for rebleeding and 30-day mortality were exploratory because of the limited number of events and require external validation. More prospective multicenter studies are needed to confirm the clinical value of the CALLY index in older individuals with UGIB.

## 1. Introduction

Upper gastrointestinal bleeding (UGIB) is a frequently encountered emergency in gastroenterology, possibly resulting in considerable morbidity and mortality. Despite substantial advances in diagnostic and therapeutic modalities, the annual incidence of UGIB remains between 50 and 180 cases per 100,000 population, with an overall mortality rate of approximately 10%. This rate is even higher in the presence of comorbid conditions [1]. UGIB constitutes a substantial healthcare issue because of prolonged hospitalizations and increased healthcare costs [2]. The increasing elderly demographic, in addition to the rising use of antiplatelet and anticoagulant medications, has led to an increasing rate in the incidence of UGIB.

In emergency situations, the prompt identification of high-risk and poor-prognosis individuals among vulnerable populations, such as the elderly, is essential for effective clinical management. Several clinical and endoscopic factors are related to adverse clinical outcomes. Age, comorbidities, presence of shock, endoscopic diagnosis, hemoglobin values at presentation, ulcers’ size, stigmata of recent hemorrhage and need for a blood transfusion have all been described as significant risk factors for rebleeding and death [3,4]. Despite the development of numerous risk scoring systems, including Glasgow-Blatchford, Rockall, ABC, AIM65, Canada–United Kingdom–Adelaide (CANUKA), MAP(ASH), and T score, their use may be time-consuming and practically limited for specific cases [5]. The majority of existing UGIB evaluation systems were established among various adult cohorts. Nonetheless, aged patients significantly vary in terms of comorbidity burden, frailty, polypharmacy, and physiological reserve, all of which may affect the prediction efficacy of various scoring systems. As a result, their precision in senior patients is still unclear.

The C-reactive protein (CRP)-albumin-lymphocyte (CALLY) index is a newly developed inflammation-based biomarker that evaluates parameters such as the serum CRP level, serum albumin level, and peripheral lymphocyte count [6]. The CALLY index has been reported to predict the prognosis of several malignancies [7,8,9]. Although its applicability as a prognostic marker has been evidenced, especially in oncological conditions like gastric cancer, its prognostic significance in upper gastrointestinal bleeding remains insufficiently verified.

The main aim of this study was to evaluate the predictive performance of established UGIB risk scoring systems for clinically important outcomes in older adults with acute UGIB and to compare the predictive performance of the inflammation-based CALLY index with conventional UGIB risk scores. The primary objective was to compare the discriminatory performance of the CALLY index and established UGIB risk scores for the primary management endpoint of endoscopic hemostatic therapy. Analyses of transfusion requirement, ICU admission, length of hospital stay, rebleeding, and 30-day mortality were considered secondary. Given the small number of rebleeding events and deaths, the analyses of rebleeding and 30-day mortality were considered exploratory and hypothesis-generating. To our knowledge, this is the first study to comprehensively compare the CALLY index with established UGIB risk scores in an exclusively geriatric population with acute UGIB.

## 2. Materials and Methods

This retrospective single-center observational study included geriatric patients (age ≥ 65) who were admitted to the emergency department with symptoms and signs of UGIB and underwent esophagogastroduodenoscopy (EGD) at our tertiary referral center between 1 January 2025, and 31 December 2025. Patients with missing data, those who presented with variceal bleeding, or patients who could not undergo EGD were excluded from the study. Patients who developed gastrointestinal bleeding during hospitalization were also excluded from the study.

Demographic data, medication history, comorbidities (atrial fibrillation, hypertension, cerebrovascular disease, chronic liver disease, diabetes mellitus, cancer history, chronic renal failure, and previous UGIB history), systolic blood pressure, diastolic blood pressure, melena/hematemesis, hemoglobin, platelets, lymphocytes, INR (international normalized ratio), albumin, CRP (C-reactive protein), urea, and creatinine levels were recorded from the electronic files of the patients.

The Glasgow–Blatchford score (GBS), Rockall, AIMS65, ABC, CALLY, T-score, CANUKA, MAP(ASH) were calculated. The CALLY index was calculated using the following formula: CALLY index = serum albumin (g/dL) × absolute lymphocyte count (×10^9^/L)/CRP (mg/L). Laboratory values were entered into the formula using these units without additional unit conversion. Laboratory parameters used to calculate the CALLY index, including C-reactive protein, serum albumin, and absolute lymphocyte count, were obtained from the first blood samples collected at hospital admission, before endoscopic evaluation and therapeutic interventions. The endoscopic diagnosis and intervention data were collected from the endoscopic examination reports. The clinical outcomes, including the need for endoscopic treatment, rebleeding, erythrocyte suspension transfusion number, need for embolization/surgery, need for intensive care unit admission, length of hospital stay, and 30-day mortality, were recorded for each patient. The requirement for endoscopic hemostatic therapy was designated as the primary management endpoint because the study specifically aimed to evaluate whether the investigated scoring systems, including the CALLY index, could discriminate patients who underwent therapeutic endoscopic intervention. However, endoscopic hemostasis was considered a management-related endpoint rather than a direct measure of bleeding severity, as the decision to perform endoscopic therapy may be influenced by lesion characteristics, endoscopic findings, and clinical judgment. All risk scores were calculated retrospectively for the purposes of the study and were not available to the treating endoscopists at the time of clinical decision-making. The mortality endpoint was defined as 30-day all-cause mortality. Patients discharged before 30 days were followed through the Turkish National Health Information System, which was used to determine survival status.

Red blood cell replacement was performed to ensure a target hemoglobin (Hb) value of 10 g/dL in patients with cardiac risk factors and 7 to 9 g/dL in other patients. A proton pump inhibitor (PPI) (8 mg/h) was started immediately after the patient was admitted to the emergency department and before the EGD examination [10]. Endoscopic hemostatic therapy was performed in patients with Forrest Ia, Ib, IIa, or IIb ulcers, as well as in patients with Mallory–Weiss tears or Dieulafoy lesions when active bleeding or high-risk endoscopic stigmata requiring endoscopic hemostasis were present. Endoscopic hemostatic techniques included hemoclip application, injection therapy, thermal coagulation, or combinations of these modalities according to the endoscopist’s judgment. EGD was performed within the first 24 h of admission.

Clinical evidence for recurrent bleeding is defined as the recurrence of hematemesis, development of tachycardia and hypotension after hemodynamic stability, development of melena and/or hematochezia after normal defecation, and a decrease in the Hb value of more than 2 g/dL [11]. For bleeding that could not be stopped after two endoscopic treatments, the patient was referred for surgery or embolization.

The study was conducted in accordance with the Declaration of Helsinki and approved by the Samsun University Non-Interventional Clinical Research Ethics Committee (Protocol/Approval No. 2026/9/18, approved on 20 May 2026).

### Statistical Analysis

Statistical analyses were performed using IBM SPSS Statistics for Windows, version 27.0 (IBM Corp., Armonk, NY, USA) and MedCalc Statistical Software, version 23.6.3 (MedCalc Software Ltd., Ostend, Belgium). Continuous variables were assessed for normality using the Shapiro–Wilk test and visual inspection of histograms. Normally distributed variables are presented as mean ± standard deviation (SD), whereas non-normally distributed variables are expressed as median and interquartile range (IQR). Categorical variables are presented as frequencies and percentages. Comparisons between patients who underwent endoscopic treatment and those who did not were performed using the independent-samples Student’s *t*-test or the Mann–Whitney *U* test for continuous variables, as appropriate. Categorical variables were compared using the chi-square test or Fisher’s exact test when expected cell counts were less than five.

For clarity, the statistical analyses were organized according to the hierarchy of study endpoints. Endoscopic hemostatic therapy was analyzed as the primary management endpoint. Transfusion requirement, ICU admission, and length of hospital stay were analyzed as secondary outcomes. Given the small number of events, the analyses of rebleeding (*n* = 8) and 30-day mortality (*n* = 7) were considered exploratory and hypothesis-generating.

The discriminatory performances of the ASA, Glasgow–Blatchford Score (GBS), AIMS65, ABC, Rockall, CALLY, Takahashi (T-score), CANUKA, and MAP(ASH) scoring systems for predicting blood transfusion requirement, endoscopic hemostatic intervention, rebleeding, ICU admission, and 30-day mortality were evaluated using receiver operating characteristic (ROC) curve analysis. Areas under the ROC curves (AUCs), with corresponding 95% confidence intervals (CIs), were calculated. For predictors inversely associated with an outcome, the direction of the ROC analysis was reversed so that higher reported AUC values consistently represented better discriminatory performance. For the CALLY mortality analysis, the ROC direction was reversed so that lower CALLY values indicated a higher probability of 30-day mortality; the AUC and its 95% CI were recalculated in the reversed direction. Optimal cutoff values were determined using the Youden index. Because these outcome-specific cutoff values were derived and evaluated within the same cohort without internal or external validation, they should be considered exploratory.

CALLY was selected as the reference score for pairwise comparisons of ROC curves. The AUC of CALLY was compared separately with those of ASA, GBS, AIMS65, ABC, Rockall, T-score, CANUKA, and MAP(ASH) for blood transfusion requirement, endoscopic hemostatic intervention, and ICU admission using the nonparametric DeLong method for correlated ROC curves. Within each clinical outcome, the resulting *p* values were adjusted for multiple comparisons using the Holm–Bonferroni method. Rebleeding and 30-day mortality were not subjected to pairwise DeLong comparisons because of the limited number of outcome events.

The association between each clinical risk score and length of hospital stay was evaluated separately using univariable generalized linear regression models with a gamma distribution and log-link function because length of hospital stay was positively skewed. For each model, regression coefficients (β), 95% CIs, *p* values, and exponentiated coefficients [exp(β)] with corresponding 95% CIs were reported. All statistical tests were two-sided, and a *p* value < 0.05 was considered statistically significant.

## 3. Results

The study included 113 geriatric patients presenting with upper GI bleeding. Among these, 42 (37.2%) underwent endoscopic hemostatic therapy and 71 (62.8%) were managed without endoscopic intervention. Baseline demographic, clinical, laboratory, and risk-score characteristics according to endoscopic treatment status are summarized in Table 1.

The mean age of the study population was 77.7 ± 8.3 years, with no significant age difference between patients who required endoscopic treatment and those who did not (75.9 ± 7.9 vs. 78.8 ± 8.3 years, *p* = 0.070). Likewise, sex distribution, presenting symptoms, hemodynamic parameters, hemoglobin level, platelet count, lymphocyte count, INR, albumin concentration, and urea levels were comparable between the two groups (all *p* > 0.05).

The most common endoscopic diagnoses were duodenal ulcer (30.1%) and gastric ulcer (29.2%), followed by other lesions (13.3%), malignancy (11.5%), Mallory–Weiss tear (5.3%), and esophageal ulcer (2.7%). No bleeding lesion was identified in 9 patients (8.0%).

Endoscopic hemostatic therapy was performed in 42 patients (37.2%), whereas surgery or radiological intervention was required in only 5 patients (4.4%). Rebleeding occurred in 8 patients (7.1%), 68 patients (60.2%) required intensive care unit admission, and the overall 30-day mortality rate was 6.2%. The median length of hospital stay was 4 (IQR, 2–7) days. Blood transfusion was required in 95 patients (84.1%), with a median of 3 (IQR, 2–5) units of packed red blood cells transfused. The clinical characteristics, endoscopic findings, and outcomes of the study cohort are summarized in Table 2.

Most patients had at least one comorbidity (89.4%). Although the overall prevalence of comorbid conditions tended to be higher in the non-endoscopic treatment group, the difference did not reach statistical significance (94.4% vs. 81.0%, *p* = 0.054). Among individual comorbidities, cerebrovascular disease was significantly more frequent in patients who did not require endoscopic treatment than in those who underwent endoscopic intervention (19.7% vs. 2.4%, *p* = 0.009). No significant differences were observed regarding diabetes mellitus, hypertension, cardiac disease, chronic kidney disease, liver cirrhosis, or malignancy.

Regarding laboratory findings, patients requiring endoscopic treatment had significantly lower CRP levels (median 5 vs. 13 mg/L, *p* = 0.002) and lower serum creatinine concentrations (median 0.96 vs. 1.37 mg/dL, *p* = 0.009), whereas the remaining laboratory parameters were similar between groups.

Comparison of risk stratification tools demonstrated significant differences in several scoring systems. Patients who underwent endoscopic treatment had lower ASA classification (median 2 vs. 3, *p* = 0.001), lower AIMS65 scores (median 1 vs. 2, *p* = 0.019), lower ABC scores (median 5 vs. 7, *p* = 0.005), lower MAP(ASH) scores (median 3 vs. 4, *p* = 0.019), and significantly higher CALLY index values (median 1.2 vs. 0.4, *p* = 0.004). In contrast, Glasgow-Blatchford, Rockall, T-score, and CANUKA scores did not differ significantly between the groups.

### 3.1. Performance of Risk Scoring Systems for Clinical Outcomes

**Figure 1:**
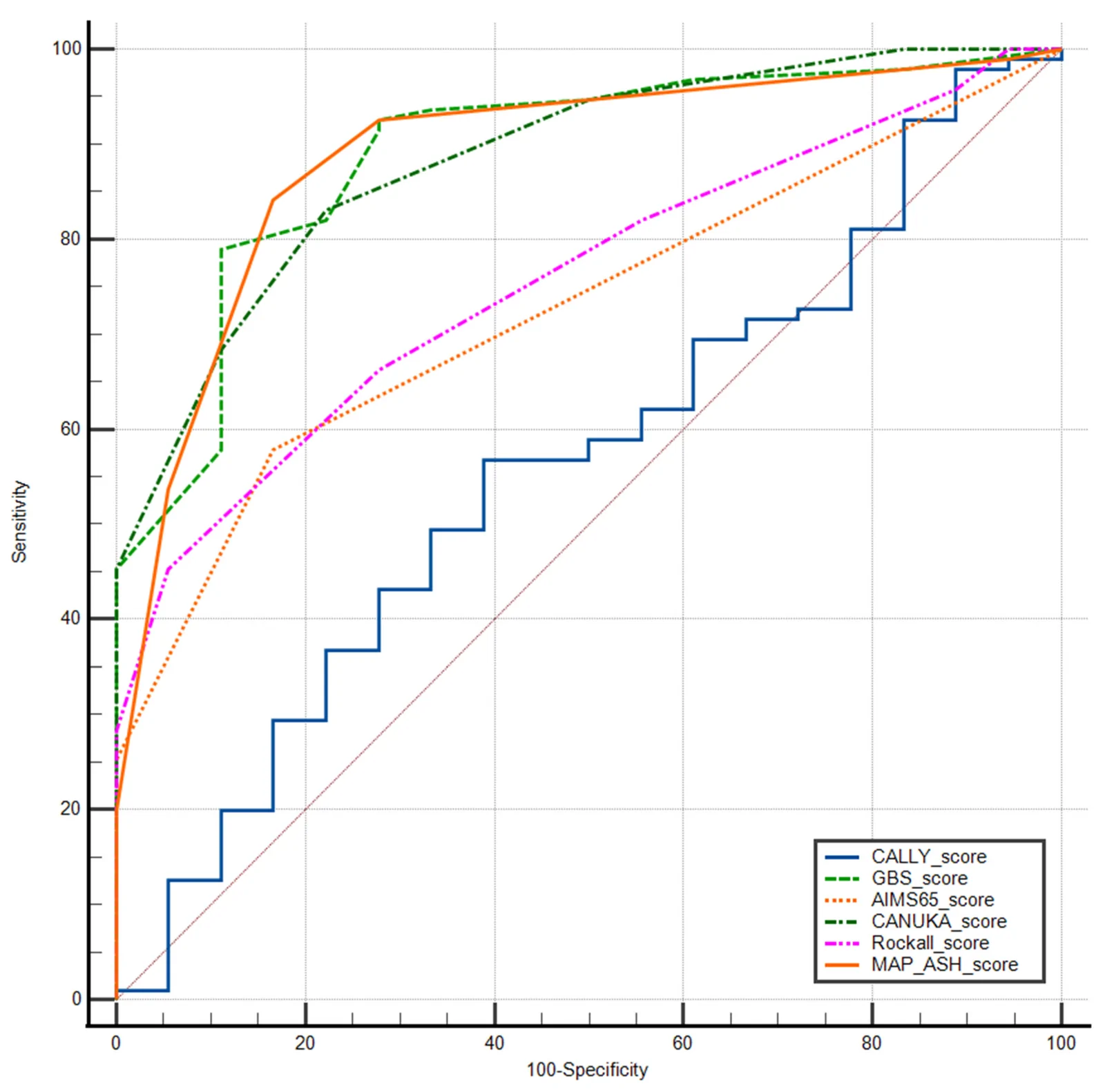
ROC curves for blood transfusion requirement.

**Figure 2:**
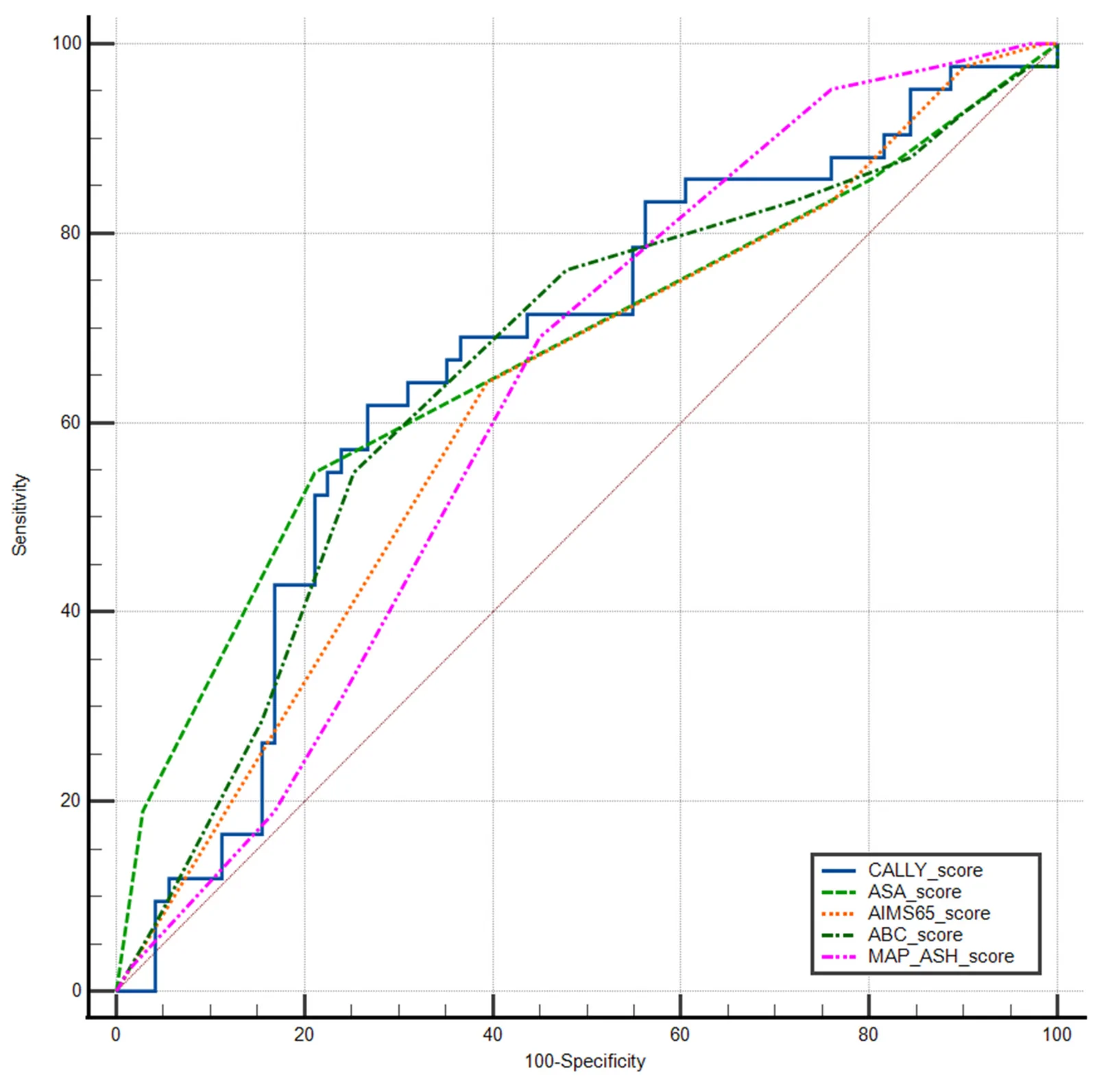
ROC curves for endoscopic intervention.

**Figure 3:**
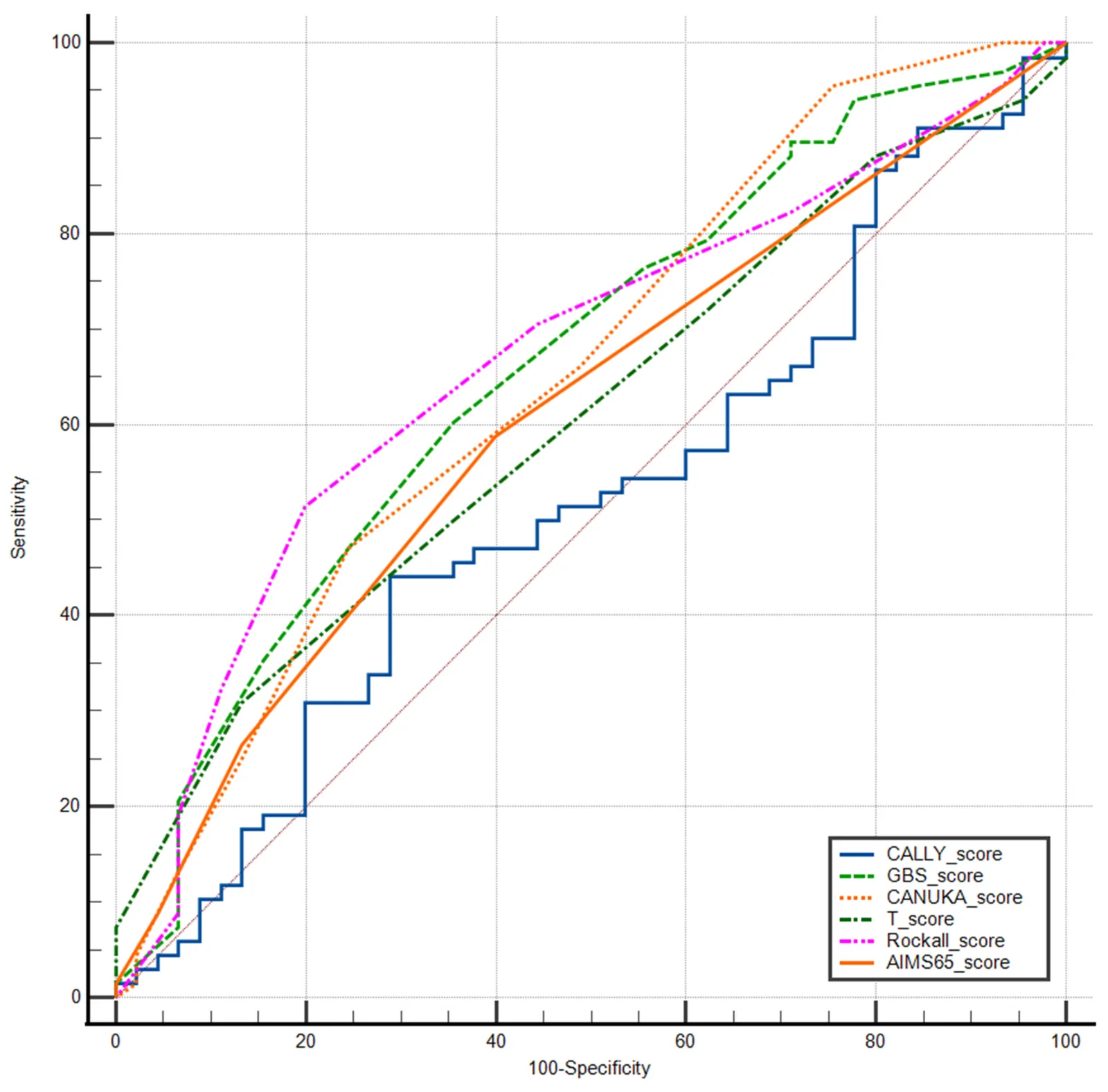
ROC intensive care unit admission.

Receiver operating characteristic (ROC) analyses were performed to evaluate the predictive performance of the investigated scoring systems for both primary and secondary clinical outcomes (Table 3). The ROC curves for blood transfusion requirement, endoscopic intervention, and ICU admission are presented in Figure 1, Figure 2 and Figure 3, respectively.

### 3.2. Prediction of Endoscopic Intervention

For the primary management endpoint of endoscopic hemostatic therapy, ASA showed the highest AUC (0.669, 95% CI: 0.560–0.778, *p* = 0.003), followed closely by the CALLY index (AUC = 0.664, 95% CI: 0.560–0.769, *p* = 0.004), ABC score (AUC = 0.655, 95% CI: 0.548–0.762, *p* = 0.006), MAP(ASH) score (AUC = 0.629, 95% CI: 0.527–0.731, *p* = 0.023), and AIMS65 score (AUC = 0.622, 95% CI: 0.515–0.729, *p* = 0.031); however, the associations for ASA, ABC, MAP(ASH), and AIMS65 were in the inverse direction.

### 3.3. Prediction of Blood Transfusion

For predicting blood transfusion requirements, the Glasgow-Blatchford Score demonstrated the highest diagnostic accuracy (AUC = 0.892, 95% CI: 0.815–0.968), closely followed by MAP(ASH) (AUC = 0.889) and CANUKA (AUC = 0.886) (all *p* < 0.001). T-score also showed excellent predictive performance (AUC = 0.851, *p* < 0.001). Rockall, AIMS65, and scores were significant but exhibited only moderate discrimination, whereas ASA, ABC, and CALLY index were not significantly associated with transfusion requirements.

### 3.4. Prediction of Rebleeding

In the exploratory analysis of rebleeding, the Rockall score had the highest observed AUC (0.739, *p* = 0.024), followed by the CALLY index (AUC = 0.724, *p* = 0.035). None of the remaining scoring systems showed statistically significant discrimination for this outcome. Because only eight rebleeding events occurred and no pairwise AUC comparisons were performed for this outcome, these findings should not be interpreted as evidence that one score was statistically superior to another and should be considered exploratory and hypothesis-generating.

### 3.5. Prediction of Intensive Care Unit Admission

For ICU admission, the Rockall score provided the highest predictive accuracy (AUC = 0.668, *p* = 0.003), followed by the Glasgow-Blatchford Score (AUC = 0.662, *p* = 0.004) and CANUKA score (AUC = 0.651, *p* = 0.007). Other scoring systems demonstrated limited or non-significant discriminative performance.

### 3.6. Prediction of 30-Day Mortality

In the exploratory analysis of 30-day mortality, based on seven deaths, the directionally corrected AUC was 0.846 for CALLY (95% CI: 0.758–0.934, *p* = 0.002), 0.756 for MAP(ASH) (*p* = 0.024), and 0.742 for CANUKA (*p* = 0.033). Lower CALLY values were associated with an increased probability of 30-day mortality, with an exploratory optimal cutoff value of ≤0.08. None of the remaining scoring systems showed statistically significant discrimination for this outcome. Because no pairwise AUC comparisons were performed for mortality, the observed differences between scores should not be interpreted as evidence of statistical superiority. These findings are exploratory and hypothesis-generating and require external validation.

### 3.7. Predictors of Hospital Length of Stay

Length-of-stay data were available for 108 of the 113 patients. Five patients were discharged after endoscopic evaluation because hospitalization was not clinically indicated and were therefore excluded from the length-of-stay analysis. Separate univariable generalized linear regression models were fitted in the remaining 108 patients to evaluate the association between each risk score and length of hospital stay (Table 4). Higher GBS (exp(β) = 1.062, 95% CI: 1.017–1.107, *p* = 0.006), AIMS65 (exp(β) = 1.339, 95% CI: 1.155–1.553, *p* < 0.001), ABC (exp(β) = 1.083, 95% CI: 1.006–1.165, *p* = 0.033), Rockall (exp(β) = 1.195, 95% CI: 1.104–1.294, *p* < 0.001), CANUKA (exp(β) = 1.142, 95% CI: 1.058–1.235, *p* < 0.001), and MAP(ASH) scores (exp(β) = 1.145, 95% CI: 1.036–1.266, *p* = 0.008) were significantly associated with a longer hospital stay. In contrast, ASA classification (*p* = 0.709), CALLY index (*p* = 0.421), and T-score (*p* = 0.266) were not significantly associated with length of hospital stay. Length-of-stay data were available for 108 of the 113 patients. Five patients were discharged after endoscopic evaluation because hospitalization was not clinically indicated and were therefore excluded from the length-of-stay analysis. Separate univariable generalized linear regression models were fitted in the remaining 108 patients to evaluate the association between each risk score and length of hospital stay (Table 4).

### 3.8. Pairwise Comparison of ROC Curves

In pairwise DeLong comparisons, CALLY showed significantly lower discriminatory performance for blood transfusion requirement than GBS (AUC difference = −0.330, Holm-adjusted *p* = 0.002), T-score (AUC difference = −0.289, Holm-adjusted *p* = 0.003), CANUKA (AUC difference = −0.324, Holm-adjusted *p* = 0.002), and MAP(ASH) (AUC difference = −0.327, Holm-adjusted *p* = 0.002). No statistically significant differences were observed between CALLY and the remaining scores for transfusion after Holm–Bonferroni correction. For endoscopic intervention and ICU admission, no statistically significant differences were observed between the AUC of CALLY and those of any of the conventional risk scores after Holm–Bonferroni correction (Table 5). Additional pairwise comparisons among the conventional risk scores are presented in Supplementary Table S1.

## 4. Discussion

The present study compared the predictive performance of established UGIB risk scoring systems and the CALLY index in a geriatric population. The principal finding was that no single scoring system consistently outperformed the others across all clinically relevant outcomes. GBS had the highest observed AUC for transfusion requirement, whereas Rockall had the highest observed AUC for rebleeding and ICU admission. However, the rebleeding finding was based on only eight events and should be considered exploratory rather than evidence of statistical superiority. In the exploratory analysis of 30-day mortality, the directionally corrected AUC was 0.846 for CALLY, compared with 0.756 for MAP(ASH) and 0.742 for CANUKA. Lower CALLY values were associated with an increased probability of 30-day mortality. However, because this analysis was based on only seven deaths and no pairwise AUC comparisons were performed, the observed AUC differences should not be interpreted as evidence of statistical superiority. In addition, higher GBS, AIMS65, ABC, Rockall, CANUKA, and MAP(ASH) scores were associated with prolonged hospitalization. After Holm–Bonferroni correction, pairwise comparisons showed that CALLY had significantly lower discriminatory performance for transfusion requirement than GBS, T-score, CANUKA, and MAP(ASH). However, its performance did not differ significantly from that of any conventional risk score for endoscopic intervention or ICU admission.

GBS, Rockall, T-score, and CANUKA scores did not demonstrate statistically significant discrimination for the need for endoscopic treatment in geriatric patients. An unexpected finding of our study was that several established risk scores, including AIMS65, ABC, and MAP(ASH), were lower in patients requiring endoscopic hemostatic therapy. Most established UGIB scores were primarily developed to predict mortality or other adverse clinical outcomes rather than the need for endoscopic treatments. In contrast, patients who did not require endoscopic therapy frequently had higher levels of systemic inflammation, renal dysfunction, and comorbidity, resulting in higher prognostic scores despite the absence of endoscopic high-risk stigmata. Importantly, the requirement for endoscopic hemostatic therapy represents a management-related endpoint rather than a direct measure of bleeding severity. Therefore, comparisons among risk scores for this endpoint should be interpreted cautiously, particularly because most established UGIB scores were not specifically developed to predict the decision to perform endoscopic hemostasis.

Ultimately, although the CALLY index was not associated with transfusion requirements or length of hospital stay, it demonstrated significant discrimination for 30-day mortality (directionally corrected AUC = 0.846, *p* = 0.002), with lower CALLY values associated with an increased risk of death. However, given the small number of deaths (*n* = 7), this finding should be considered exploratory and hypothesis-generating and requires external validation. The CALLY index also showed modest discriminatory ability for predicting the need for endoscopic hemostatic therapy (AUC = 0.664, *p* = 0.004). After Holm–Bonferroni correction, pairwise comparisons showed no statistically significant differences between the AUC of CALLY and those of any of the conventional risk scores for this outcome. However, these findings should be interpreted cautiously, as several conventional risk scores demonstrated significant associations with endoscopic intervention in the opposite direction from that expected based on their conventional risk-stratification frameworks.

Patients who underwent endoscopic therapy had lower CRP levels and fewer comorbid conditions, which may have contributed to higher CALLY values despite the presence of endoscopically treatable bleeding lesions. Conversely, patients who did not undergo endoscopic hemostasis had a greater inflammatory burden and higher comorbidity prevalence, which may have contributed to lower CALLY values. These findings indicate that the CALLY index may primarily reflect the patient’s systemic inflammatory and nutritional status rather than bleeding severity alone. Therefore, CALLY may provide complementary information when interpreted alongside established UGIB risk scores, rather than serving as a standalone predictor of bleeding-related outcomes.

Duodenal ulcers (17–37%) and gastric ulcers (11–24%) are the most common causes of UGIB [12,13]. In our study, the most frequent causes of bleeding were duodenal ulcer (30.1%) and gastric ulcer (29.2%). The average length of hospital stay due to UGIB in 120,835 geriatric patients was 4.8 days [14]. In our series, the median length of hospital stay was 4 days. In a study of 492 patients over the age of 60, the 30-day mortality rate was found to be 8.9%. [15]. Similarly, our study found a mortality rate of 6.2%. These findings were consistent with studies in the literature.

Wang et al. discovered a positive linear association between the clinical Rockall scores and patient outcomes, including rebleeding, surgery, and mortality, in 341 elderly patients with UGIB [16]. In a study conducted with 335 geriatric patients, Kalkan et al. showed that the Rockall score was superior to GBS and AIMS65 in rebleeding and mortality. However, in terms of transfusion needs and hospital stays, GBS was superior to Rockall and AIMS65 [17]. In a study comparing GBS, T-score, MAP(ASH), CANUKA, and AIMS65 scores in 136 patients, no effect of scoring systems was observed on the need for red blood transfusions, endoscopic treatment, rebleeding, and 30-day mortality [18]. In a study examining 315 peptic ulcer bleeding cases, the AIMS65 and ABC scoring systems are more effective in predicting in-hospital mortality in geriatric patients compared to the GBS and T-score [19].

The CALLY index is calculated using the serum CRP level, serum albumin level, and peripheral blood lymphocyte count, representing the inflammatory, nutritional, and immunological statuses of the patient. Serum albumin is a multifactorial biomarker influenced by nutritional status, systemic inflammation, liver function, chronic illness, and intravascular fluid status. In patients with acute UGIB, serum albumin should therefore be interpreted as an integrated marker of overall physiological condition rather than as an isolated indicator of nutritional status. CRP is an acute phase protein that is generated by inflammatory cytokines, such as interleukin-6 (IL-6) [20]. The CALLY index was originally introduced in patients with hepatocellular carcinoma, in whom a low index was significantly associated with worse survival following hepatectomy [9]. In gastric cancer, Sakurai et al. identified the CALLY index as an independent predictor of major postoperative complications [21]. Zhang et al. demonstrated that lower CALLY index values were independently associated with increased 30-day mortality in sepsis patients [22]. When taken as a whole, these studies show that the CALLY index represents nutritional reserve and systemic inflammation in a wide range of illnesses. However, there is still very little information about its involvement in acute gastrointestinal bleeding.

To our knowledge, this is the first study to compare the CALLY index with established UGIB risk scoring systems specifically in a geriatric population. The principal findings of the present study are as follows: the CALLY index demonstrated modest discriminatory performance for predicting the need for endoscopic hemostatic therapy and showed significant discrimination for rebleeding and 30-day mortality, whereas conventional scoring systems such as GBS, MAP(ASH), and CANUKA remained useful for predicting transfusion requirements and other clinical outcomes. Given the small number of rebleeding events (*n* = 8) and deaths (*n* = 7), however, the findings for these two outcomes should be considered exploratory and hypothesis-generating and require external validation. Implementing scoring systems that determine risk classification, which include many parameters, in the emergency department can be challenging. The use of these scoring systems may be limited in geriatric patients. Because the CALLY index is derived from routinely available laboratory parameters, it may represent a practical inflammation-based marker that warrants further evaluation in combination with established UGIB risk scores. Our findings should be interpreted as a comparison of predictive performance rather than evidence of incremental prognostic value beyond established risk scores.

The most significant limitation of the study is that it was conducted in a single center with a low number of patients. The small number of outcome events, particularly for rebleeding (*n* = 8) and 30-day mortality (*n* = 7), limits the statistical precision and robustness of the ROC analyses for these endpoints. Therefore, these findings should be considered exploratory and interpreted with caution until validated in larger prospective cohorts. Because of the limited sample size and the low number of outcome events, particularly for mortality and rebleeding, formal analyses of incremental predictive value (e.g., multivariable model updating, likelihood-ratio testing, calibration assessment, or decision-curve analysis) were not performed. Therefore, the present study should be interpreted as a comparative evaluation of predictive performance rather than a demonstration of incremental prognostic value. In addition, because only patients who underwent upper gastrointestinal endoscopy were included, the study population may not fully represent all geriatric patients presenting with acute UGIB. Patients considered too frail or hemodynamically unstable to undergo endoscopy may have been underrepresented, introducing potential selection bias. Because the full Rockall score incorporates endoscopic findings, its performance for predicting the need for endoscopic intervention should be interpreted with caution and is not directly comparable with purely pre-endoscopic risk scores. Because management-related outcomes were influenced by institutional practice patterns and clinical decision-making, these endpoints may not exclusively reflect disease severity. Therefore, the predictive performance of the evaluated scoring systems for these outcomes may not be fully generalizable to other healthcare settings. Moreover, the absence of multivariable modelling should be considered a major limitation of this study. Consequently, the observed associations may have been influenced by residual confounding, and the findings should be interpreted as hypothesis-generating rather than evidence of independent prognostic value.

## 5. Conclusions

No single risk scoring system accurately predicted all clinically relevant outcomes in older adults with acute UGIB. Established UGIB risk scores demonstrated variable performance depending on the clinical endpoint, with GBS, Rockall, MAP(ASH), and CANUKA remaining useful for specific outcomes. The CALLY index showed modest discrimination for endoscopic hemostatic therapy. Associations with rebleeding and 30-day mortality were also observed; however, because these analyses were based on only eight rebleeding events and seven deaths, respectively, the findings should be considered exploratory and hypothesis-generating and require external validation. These findings suggest that the CALLY index may serve as an inflammation-based marker for risk stratification in geriatric patients when interpreted alongside established UGIB risk scores. However, the present study was designed to compare predictive performance rather than to evaluate the incremental prognostic value of the CALLY index beyond existing models. Larger prospective multicenter studies are warranted to validate these findings and to determine the potential clinical utility of incorporating the CALLY index into future risk prediction models.

## Figures and Tables

**Table 1:** Baseline demographic and clinical characteristics of patients according to endoscopic treatment.

Variables	No Endoscopic Treatment (*n* = 71)	Endoscopic Treatment *(n* = 42)	Overall Cohort (*n* = 113)	*p*
Age, *mean ± SD*	78.8 ± 8.3	75.9 ± 7.9	77.7 ± 8.3	0.070
Sex, *n* (%) Male Female	39 (54.9) 32 (45.1)	28 (66.7) 14 (33.3)	67 (59.3) 46 (40.7)	0.220
Comorbidity present, *n* (%)	67 (94.4)	34 (81.0)	101 (89.4)	0.054 *
Diabetes mellitus, *n* (%)	23 (32.4)	11 (26.2)	34 (30.1)	0.487
Hypertension, *n* (%)	57 (80.3)	30 (71.4)	87 (77.0)	0.280
Cardiac disease, *n* (%)	44 (62.0)	19 (45.2)	63 (55.8)	0.083
Cerebrovascular disease, *n* (%)	14 (19.7)	1 (2.4)	15 (13.3)	**0.009**
Liver cirrhosis, *n* (%)	1 (1.4)	4 (9.5)	5 (4.4)	0.063 *
Chronic kidney disease, *n* (%)	15 (21.1)	5 (11.9)	20 (17.7)	0.215
Cancer, *n* (%)	9 (12.7)	7 (16.7)	16 (14.2)	0.557
Heart rate, bpm, *mean ± SD*	100 (88–120)	90 (75–110)	90 (83–115)	0.052
Systolic blood pressure, mmHg, *median (IQR)*	110 (90–120)	110 (100–122)	110 (97–120)	0.589
Diastolic blood pressure, mmHg, *median (IQR)*	60 (51–73)	65 (60–70)	60 (58–70)	0.557
Melena, *n* (%)	52 (73.2)	34 (81.0)	86 (76.1)	0.353
Hematemesis, *n* (%)	23 (32.4)	15 (35.7)	38 (33.6)	0.718
Hemoglobin, g/dL, *mean ± SD*	8.0 ± 2.4	8.1 ± 2.4	8.1 ± 2.4	0.814
Platelets, 10^9^/L, *median (IQR)*	241 (195–335)	222 (179–276)	235 (185–305)	0.113
Lymphocytes, 10^9^/L, *median (IQR)*	1.52 (1.21–2.04)	1.70 (1.20–2.16)	1.58 (1.21–2.09)	0.343
INR, *median (IQR)*	1.11 (1.03–1.41)	1.09 (1.03–1.17)	1.10 (1.03–1.26)	0.168
Albumin, g/dL, *median (IQR)*	3.1 (2.9–3.5)	3.2 (2.9–3.5)	3.2 (2.9–3.5)	0.489
CRP, mg/L, *median (IQR)*	13 (5–71)	5 (3–17)	9 (4–57)	**0.002**
Urea, mg/dL, *median (IQR)*	93 (61–135)	95 (57–138)	94 (61–133)	0.908
Creatinine, mg/dL, *median (IQR)*	1.37 (1.00–1.87)	0.96 (0.82–1.31)	1.20 (0.88–1.72)	**0.009**
Risk scores, *median (IQR)* ASA GBS AIMS65 ABC Rockall CALLY T-score CANUKA MAP(ASH)	3 (3–3) 13 (11–16) 2 (1–2) 7 (5–8) 6 (5–7) 0.4 (0.1–0.9) 8 (6–9) 11 (9–12) 4 (3–4)	2 (2–3) 14 (11–16) 1 (1–2) 5 (4–6) 7 (6–8) 1.2 (0.3–2.3) 8 (7–9) 11 (9–12) 3 (2–4)	3 (2–3) 14 (11–16) 2 (1–2) 6 (5–7) 6 (5–7) 0.6 (0.1–1.6) 8 (7–9) 11 (9–12) 3 (2–4)	**0.001** 0.919 **0.019** **0.005** 0.053 **0.004** 0.286 0.624 **0.019**

**Abbreviations:** ABC, Age, Blood tests, and Comorbidities score; AIMS65, Albumin, International Normalized Ratio, Mental status, Systolic blood pressure, and Age ≥65 years; ASA, American Society of Anesthesiologists physical status classification; CALLY, C-reactive protein–Albumin–Lymphocyte index; CANUKA, Canadian Upper Gastrointestinal Bleeding score; CRP, C-reactive protein; GBS, Glasgow-Blatchford Score; INR, International Normalized Ratio; IQR, interquartile range; MAP(ASH), Mental status, ASA class, Pulse, Albumin, Systolic blood pressure, Hemoglobin score; SD, standard deviation. * Fischer’s exact test was used. Bold values indicate statistical significance (*p* < 0.05).

**Table 2:** Clinical characteristics, endoscopic findings, and clinical outcomes of the study cohort.

Variables	Overall Cohort (*n* = 113)
Hospitalization duration, days, median (IQR)	4 (2–7)
Endoscopic diagnosis, *n* (%) Gastric ulcer Duodenal ulcer Esophageal ulcer Mallory-Weiss Malignancy Other No lesion identified	33 (29.2) 34 (30.1) 3 (2.7) 6 (5.3) 13 (11.5) 15 (13.3) 9 (8.0)
Blood transfusion, *n* (%)	95 (84.1)
Units of packed red blood cells transfused, median (IQR)	3 (2–5)
Endoscopic intervention, *n* (%)	42 (37.2)
Surgery/radiological intervention, *n* (%)	5 (4.4)
Rebleeding, *n* (%)	8 (7.1)
Intensive care unit admission, *n* (%)	68 (60.2)
30-day mortality, *n* (%)	7 (6.2)

**Abbreviations:** IQR, interquartile range.

**Table 3:** ROC analysis of scoring systems for clinical outcomes in geriatric patients with upper gastrointestinal bleeding.

**Panel A. Blood Transfusion Requirement (*n* = 95)**					
**Score**	**AUC (95% CI)**	***p***	**Cut-Off**	**Sensitivity**	**Specificity**
ASA	0.591 (0.437–0.745)	0.221	≥2.5	69.5	50.0
GBS	0.892 (0.815–0.968)	**<0.001**	**≥10.5**	91.6	72.2
AIMS65	0.727 (0.622–0.833)	**0.002**	**≥1.5**	57.9	83.3
ABC	0.555 (0.412–0.698)	0.461	≥6.5	43.2	66.7
Rockall	0.757 (0.656–0.858)	**0.001**	**≥5.5**	66.3	72.2
CALLY	0.562 (0.420–0.704)	0.406	≥0.43	56.8	55.6
T-score	0.851 (0.777–0.925)	**<0.001**	**≤8.5**	77.9	83.3
CANUKA	0.886 (0.812–0.959)	**<0.001**	**≥9.5**	83.2	77.8
MAP(ASH)	0.889 (0.807–0.971)	**<0.001**	**≥2.5**	84.2	83.3
**Panel B. Endoscopic Intervention (*n* = 42)**					
**Score**	**AUC (95% CI)**	***p***	**Cut-Off**	**Sensitivity**	**Specificity**
ASA	0.669 (0.560–0.778)	**0.003**	**≤2.5**	54.8	78.9
GBS	0.506 (0.396–0.616)	0.920	≥13.5	54.8	52.1
AIMS65	0.622 (0.515–0.729)	**0.031**	**≤1.5**	64.3	60.6
ABC	0.655 (0.548–0.762)	**0.006**	**≤** **5.5**	54.8	76.4
Rockall	0.608 (0.498–0.718)	0.056	≥5.5	76.2	49.3
CALLY	0.664 (0.560–0.769)	**0.004**	**≥1.62**	38.1	83.1
T-score	0.559 (0.450–0.668)	0.294	≥7.5	61.9	47.9
CANUKA	0.527 (0.418–0.636)	0.628	**≤**11.5	69.9	42.3
MAP(ASH)	0.629 (0.527–0.731)	**0.023**	**≤3.5**	69.0	54.9
**Panel C. Rebleeding (*n* = 8)**					
**Score**	**AUC (95% CI)**	***p***	**Cut-Off**	**Sensitivity**	**Specificity**
ASA	0.590 (0.382–0.798)	0.398	**≤2.5**	50.0	67.6
GBS	0.665 (0.480–0.849)	0.121	≥11.5	100.0	29.5
AIMS65	0.539 (0.308–0.770)	0.716	≥2.5	37.5	80.0
ABC	0.557 (0.366–0.748)	0.591	**≤**6.5	75.0	42.9
Rockall	0.739 (0.588–0.890)	**0.024**	**≥6.5**	75.0	63.8
CALLY	0.724 (0.579–0.868)	**0.035**	**≥2.1**	37.5	81.0
T-score	0.504 (0.312–0.695)	0.973	**≤**8.5	75.0	32.4
CANUKA	0.592 (0.413–0.770)	0.389	≥11.5	62.5	63.8
MAP(ASH)	0.529 (0.404–0.653)	0.788	**≤**3.5	75.0	47.6
**Panel D. ICU Admission (*n* = 68)**					
**Score**	**AUC (95% CI)**	***p***	**Cut-Off**	**Sensitivity**	**Specificity**
ASA	0.527 (0.418–0.635)	0.631	**≤**1.5	11.8	95.6
GBS	0.662 (0.560–0.765)	**0.004**	**≥13.5**	60.3	64.4
AIMS65	0.608 (0.503–0.713)	0.052	≥1.5	58.8	60.0
ABC	0.522 (0.414–0.631)	0.688	**≤**6.5	61.8	46.7
Rockall	0.668 (0.567–0.770)	**0.003**	**≥5.5**	70.6	55.6
CALLY	0.517 (0.408–0.626)	0.756	**≤**0.41	47.1	62.2
T-score	0.605 (0.501–0.709)	0.059	**≤**6.5	30.9	86.7
CANUKA	0.651 (0.547–0.756)	**0.007**	**≥10.5**	66.2	51.1
MAP(ASH)	0.555 (0.444–0.667)	0.320	≥2.5	80.9	37.8
**Panel E. 30-Day Mortality (*n* = 7)**					
**Score**	**AUC (95% CI)**	***p***	**Cut-Off**	**Sensitivity**	**Specificity**
ASA	0.630 (0.438–0.822)	0.250	≥2.5	85.7	34.9
GBS	0.649 (0.415–0.883)	0.188	≥16.5	57.1	87.7
AIMS65	0.703 (0.522–0.884)	0.073	≥1.5	85.7	50.9
ABC	0.709 (0.527–0.891)	0.065	≥5.5	85.7	37.7
Rockall	0.700 (0.514–0.886)	0.077	≥5.5	85.7	41.5
CALLY	0.846 (0.758–0.934)	**0.002**	≤0.08 *	85.7	77.4
T-score	0.706 (0.456–0.955)	0.069	**≤**6.5	71.4	79.2
CANUKA	0.742 (0.537–0.946)	**0.033**	**≥11.5**	71.4	64.2
MAP-ASH	0.756 (0.536–0.976)	**0.024**	**≥3.5**	85.7	56.6

**Abbreviations:** ABC, Age, Blood tests, and Comorbidities score; AIMS65, Albumin, International Normalized Ratio, Altered Mental Status, Systolic Blood Pressure, and Age ≥65 years; ASA, American Society of Anesthesiologists physical status classification; AUC, area under the receiver operating characteristic curve; CALLY, C-reactive protein–Albumin–Lymphocyte index; CANUKA, Canada–United Kingdom–Adelaide; CI, confidence interval; GBS, Glasgow-Blatchford Score; ICU, intensive care unit; MAP(ASH), Mental status, ASA class, Pulse, Albumin, Systolic blood pressure, Hemoglobin score; * ROC, receiver operating characteristic. ROC analysis of the evaluated scoring systems for clinical outcomes. AUC, area under the receiver operating characteristic curve; CI, confidence interval. AUC values were reported according to the direction of the ROC analysis, with higher AUC values indicating better discriminatory performance. Bold values indicate statistical significance (*p* < 0.05).

**Table 4:** Univariable generalized linear regression analysis of scoring systems for length of hospital stay (*n* = 108).

Score	β Coefficient (95% Cl)	exp(β) (95% CI)	*p*
ASA	0.083 (−0.164 to 0.241)	1.087 (0.849–1.273)	0.709
GBS	0.060 (0.017 to 0.102)	1.062 (1.017–1.107)	0.006
AIMS65	0.292 (0.144 to 0.440)	1.339 (1.155–1.553)	<0.001
ABC	0.080 (0.006 to 0.153)	1.083 (1.006–1.165)	0.033
Rockall	0.178 (0.099 to 0.258)	1.195 (1.104–1.294)	<0.001
CALLY	−0.020 (−0.069 to 0.029)	0.980 (0.933–1.029)	0.421
T-score	−0.055 (−0.151 to 0.042)	0.947 (0.860–1.043)	0.266
CANUKA	0.133 (0.056 to 0.211)	1.142 (1.058–1.235)	<0.001
MAP(ASH)	0.135 (0.035 to 0.236)	1.145 (1.036–1.266)	0.008

**Abbreviations:** ASA, American Society of Anesthesiologists physical status classification; GBS, Glasgow-Blatchford Score; AIMS65, Albumin, International Normalized Ratio, Altered Mental Status, Systolic Blood Pressure, and Age ≥65 years; ABC, Age, Blood tests, and Comorbidities score; CALLY, C-reactive protein–Albumin–Lymphocyte index; CANUKA, Canada–United Kingdom–Adelaide score; MAP(ASH), Mental status, ASA class, Pulse, Albumin, Systolic Blood Pressure, and Hemoglobin score; β, beta coefficient; exp(β), exponentiated beta coefficient; CI, confidence interval. A generalized linear model with gamma distribution and log-link function was used.

**Table 5:** Pairwise comparison of ROC curves for CALLY and other clinical risk scores for blood transfusion requirement, endoscopic intervention, and ICU admission using the DeLong method.

**Panel A. Blood Transfusion Requirement (Positive *n* = 95; Total *N* = 113)**					
**Comparison**	**CALLY AUC**	**Comparator AUC**	AUC Difference *	**Raw *p***	**Holm-Adjusted *p***
CALLY vs. ASA	0.562	0.591	−0.0292	0.785	1.000
CALLY vs. GBS	0.562	0.892	−0.3300	0.001	**0.002**
CALLY vs. AIMS65	0.562	0.727	−0.1650	0.082	0.411
CALLY vs. ABC	0.562	0.555	+0.0070	0.952	1.000
CALLY vs. Rockall	0.562	0.757	−0.1950	0.035	0.140
CALLY vs. T-score	0.562	0.851	−0.2890	0.001	**0.003**
CALLY vs. CANUKA	0.562	0.886	−0.3240	0.001	**0.002**
CALLY vs. MAP(ASH)	0.562	0.889	−0.3270	0.001	**0.002**
**Panel B. Endoscopic Intervention (Positive *n* = 42; Total *N* = 113)**					
CALLY vs. ASA	0.664	0.669	−0.00453	0.944	1.000
CALLY vs. GBS	0.664	0.506	0.159	0.055	0.422
CALLY vs. AIMS65	0.664	0.622	0.0423	0.505	1.000
CALLY vs. ABC	0.664	0.655	0.00889	0.887	1.000
CALLY vs. Rockall	0.664	0.608	0.0565	0.505	1.000
CALLY vs. T-score	0.664	0.559	0.105	0.172	1.000
CALLY vs. CANUKA	0.664	0.527	0.137	0.053	0.422
CALLY vs. MAP-ASH	0.664	0.629	0.0355	0.604	1.000
**Panel C. ICU Admission (Positive *n* = 68; Total *N* = 113)**					
CALLY vs. ASA	0.517	0.527	−0.00948	0.906	1.000
CALLY vs. GBS	0.517	0.662	−0.145	0.052	0.363
CALLY vs. AIMS65	0.517	0.608	−0.0908	0.155	0.773
CALLY vs. ABC	0.517	0.522	−0.00507	0.955	1.000
CALLY vs. Rockall	0.517	0.668	−0.151	0.037	0.296
CALLY vs. T-score	0.517	0.605	−0.0879	0.251	1.000
CALLY vs. CANUKA	0.517	0.651	−0.134	0.058	0.363
CALLY vs. MAP(ASH)	0.517	0.555	−0.0381	0.612	1.000

* AUC difference = CALLY AUC − comparator AUC. Holm–Bonferroni correction was applied separately within each clinical outcome across the eight pairwise comparisons. Bold values indicate statistical significance (*p* < 0.05).

## Data Availability

The datasets used and/or analyzed in this study are available upon reasonable request from the corresponding author.

## Supplementary Materials

The following supporting information can be downloaded at: https://www.mdpi.com/article/10.3390/diagnostics16162664/s1, Table S1: Pairwise comparisons of ROC curves among established risk scores for blood transfusion requirement, endoscopic intervention, and ICU admission.

## Abbreviations

ABC	Age, Blood Tests, and Comorbidities Score
AIMS65	Albumin, International Normalized Ratio, Altered Mental Status, Systolic Blood Pressure, and Age ≥65 Years
ASA	American Society of Anesthesiologists Physical Status Classification
AUC	Area Under the Receiver Operating Characteristic Curve
CALLY	C-Reactive Protein–Albumin–Lymphocyte Index
CANUKA	Canada–United Kingdom–Adelaide
CI	Confidence Interval
CRP	C-Reactive Protein
EGD	Esophagogastroduodenoscopy
GBS	Glasgow-Blatchford Score
GI	Gastrointestinal
Hb	Hemoglobin
ICU	Intensive Care Unit
IL-6	Interleukin-6
INR	International Normalized Ratio
IQR	Interquartile Range
MAP(ASH)	Mental Status, ASA Class, Pulse, Albumin, Systolic Blood Pressure, Hemoglobin Score
PPI	Proton Pump Inhibitor
ROC	Receiver Operating Characteristic
SD	Standard Deviation
UGIB	Upper Gastrointestinal Bleeding
