# Supplementary material for: Comparative Predictive Performance of Upper Gastrointestinal Bleeding Risk Scores and the CALLY Index in Geriatric Patients
Source: Diagnostics (Basel). 2026 Aug 20;16(16):2664. doi: 10.3390/diagnostics16162664 (PMC13512241; doi:10.3390/diagnostics16162664)
Supplement: Supplementary file 1 [file diagnostics-16-02664-s001.zip › diagnostics-4471465-supplementary.pdf]

Supplementary Table S1. Pairwise comparisons of ROC curves among established risk scores for blood transfusion requirement, endoscopic intervention, and ICU admission.

Panel A. Blood transfusion requirement (*n*=95)

	CANUKA	AIMS65	Rockall	GBS	MAP(ASH)
**CANUKA**	—	0.158 (0.001)	0.129 (0.019)	0.006 (0.863)	0.004 (0.920)
**AIMS65**		—	0.030 (0.673)	0.164 (0.005)	0.162 (0.009)
**Rockall**			—	0.135 (0.026)	0.132 (0.027)
**GBS**				—	0.002 (0.919)
**MAP(ASH)**					—

Panel B. Endoscopic intervention (*n*=42)

	CANUKA	AIMS65	Rockall	GBS	MAP(ASH)
**CANUKA**	—	0.095 (0.094)	0.081 (0.396)	0.022 (0.829)	0.101 (0.009)
**AIMS65**		—	0.014 (0.866)	0.116 (0.216)	0.007 (0.907)
**Rockall**			—	0.102 (0.122)	0.021 (0.813)
**GBS**				—	0.123 (0.194)
**MAP(ASH)**					—

Panel C. ICU Admission (*n*=68)

	CANUKA	AIMS65	Rockall	GBS	MAP(ASH)
**CANUKA**	—	0.043 (0.405)	0.017 (0.758)	0.011 (0.813)	0.096 (0.017)
**AIMS65**		—	0.060 (0.298)	0.054 (0.281)	0.053 (0.341)
**Rockall**			—	0.006 (0.921)	0.113 (0.060)
**GBS**				—	0.107 (0.026)
**MAP(ASH)**					—

*Cells show p values from pairwise DeLong comparisons between AUCs. Holm–Bonferroni correction was applied for multiple comparisons within each panel.
